# Tunable Memristic Characteristics Based on Graphene Oxide Charge-Trap Memory

**DOI:** 10.3390/mi10020151

**Published:** 2019-02-23

**Authors:** Lei Li

**Affiliations:** 1 Key Laboratories of Senior-Education for Electronic Engineering, Heilongjiang University, Harbin 150080, China; 2 Research Center for Fiber Optic Sensing Technology National Local Joint Engineering, Heilongjiang University, Harbin 150080, China

**Keywords:** bipolar memristic behavior, fluorescence quench, GO charge-trap, PMMA:GO nanocomposite

## Abstract

Solution-processable nonvolatile memory devices, consisted of graphene oxide (GO) embedded into an insulating polymer polymethyl methacrylate (PMMA), were manufactured. By varying the GO content in PMMA nanocomposite films, the memristic conductance behavior of the Ni/PMMA:GO/Indium tin oxide (ITO) sandwiched structure can be tuned in a controllable manner. An investigation was made on the memristic performance mechanism regarding GO charge-trap memory; these blends were further characterized by transmission electron microscope (TEM), scanning electron microscope (SEM), Fourier transform infrared spectra (FTIR), Raman spectra, thermogravimetric analysis, X-ray diffraction (XRD), ultraviolet-visible spectroscopy, and fluorescence spectra in particular. Dependent on the GO content, the resistive switching was originated from the charges trapped in GO, for which bipolar tunable memristic behaviors were observed. PMMA:GO composites possess an ideal capability for large area device applications with the benefits of superior electronic properties and easy chemical modification.

## 1. Introduction

Resistive Random Access Memory (ReRAM), as a disruptive technology, can be compatible with conventional semiconductor processes, attracting much attention [1,2]. It can revolutionize the product performance in digital memories, particularly capable of substituting all current up-to-date memories like hard disk drives, random-access memories, and Flash memories. Among all the technology candidates, memristor-based ReRAM operates faster than phase change random access memory (PCRAM), and it possesses not a simpler, but a smaller cell structure than magnetic random access memory (MRAM) or shared transistor technology random access memory (STT-RAM) [3,4,5]. Confronted with a traditional Metal-Oxide-Semiconductor (MOS)-accessed memory cell, memristor-based RRAM bears the promising potential of forming a cross-point structure without access devices, for the sake of achieving an ultra-high density form of data storage. That is one of the emerging memory technologies, with a two-terminal metal-insulator-metal (MIM) configuration. Induced by applying different voltages to the device terminals, it utilizes functional materials switching among more than two distinct resistance states. Based on memristors where migrating oxygen atoms give rise to changable resistance, metal oxide nanolayers are investigated by many companies, shifted by voltage pulses, with the fact that the electric field leads to conducting filaments through an insulating oxide. Relying on the bistable resistance states, this device can be used with nearly any oxide material, such as NiO, ZnO, ZrO_2_, HfO_2_, SrZrO_3_, and BaTiO_3_ [6,7,8,9,10].

Confronted with theoretical and physical restrictions for conventional Si-based memory devices, a great number of efforts have been made to exploit novel functional materials for remarkable resistive switching behaviors in future memristors. Doping carbon-based nanomaterials as charge acceptors into organic matrix has attracted considerable interest for memristors [11,12,13,14,15]. The design and synthesis of a solution-processable polymer nanocomposite with tunable doping levels, predominantly desirable for memory device applications, can not only tailor the electronic memristor characteristics but also acquire excellent chemical, mechanical, and biocompatible properties. An ultrathin 2D-layered material graphene oxide (GO) plays a crucial role in developing electronic devices on the basis of atomically thin films. The chemical structure of GO has carbonyl and carboxyl groups at the edge of the plane. Doping levels of GO in the polymer nanocomposites will have a crucial influence on the charge transport processes in the bulk and at the interface, because of the excellent electronic properties of graphene, and this will consequently impact on memristic performance [16,17,18,19,20,21]. The electrical properties of GO-based nanocomposite films can be regulated by the amount of oxygen-functional groups that are attached to GO sheets, so that GO-based hybrid films can act as a remarkable active layer in memristors.

Herein, GO charge-trap memory devices, which are composed of polymethyl methacrylate (PMMA) blended with GO, were demonstrated by both simple and cost-effective solution-processable techniques at relatively low temperature. Although the memristic behaviors can be tunable by the GO content, we focus on the operating mechanism, as well as the influence of GO on the device performance. The prepared PMMA:GO nanocomposites were analyzed by means of transmission electron microscope (TEM), scanning electron microscope (SEM), Fourier transform infrared spectra (FTIR), Raman spectra, thermogravimetric analysis (TGA), X-ray diffraction (XRD), ultraviolet-visible spectroscopy, and fluorescence spectra. The principle for the memristic characteristics based on the GO charge trap was represented by fluorescent quenching.

## 2. Materials and Methods 

### 2.1. Preparation of PMMA:GO Composites

PMMA was purchased from ARKEMA, Shanghai, China, for which the molecular weight *M*_w_ ranges from 120,000 to 150,000. The purity of GO (purchased from Hengqiu Tech. Inc., Suzhou, China) was more than 90 wt %, and the thickness and diameter ranged from 3.4 nm to 7 nm and 10 μm to 50 μm, respectively. The number of graphene sheets was restrained to at most 10 layers. In different weight ratios, PMMA and GO were mixed up and dissolved into anhydrous *N*,*N*-dimethylformamide (DMF), and then PMMA:GO composites were prepared by ultrasonication in DMF for 24 h at room temperature. Then, a highly homogeneous black dispersion of PMMA:GO nanocomposites (10 mg/mL) was obtained.

### 2.2. Manufacturing of PMMA:GO Memory

Glass substrates (dimensions of 1 × 2 cm^2^) coated with indium-tin-oxide (ITO, conductivity of 10 Ω/sq) were precleaned by ultrasonication for 20 min in acetone and methanol, together with ethanol solvents. After that, the above DMF solution of the PMMA:GO hybrid was spin-coated onto ITO substrates at 3000 rpm for 60 s. Subsequently, the solvent was removed in a vacuum chamber at 10^−5^ Torr of pressure, and the samples were kept at 80 °C overnight to obtain 20 nm thick nanocomposite films. With a shallow mask, Nickel (Ni) (about 300 nm) was thermally evaporated to form the top Ni electrode at 1 × 10^−5^ Torr of pressure. 

### 2.3. Apparatus and Methods

Micrographs of GO sheets were acquired by TEM (JEM-2100, JOEL, Tokyo, Japan). An Apreo scanning electron microscope (SEM, Themoscientific, Waltham, MA, USA) was utilized to test the cross-section of the PMMA:GO hybrid films. The thickness of the nano-hybrid films was measured by NanoMap 500LS 3D Profilometer (aep Technology, Santa Clara, CA, USA). A Foss DS 2500 infrared spectrometer (Foss NIRS DS, Munkedal, Sweden) was adopted to measure the FTIR of the PMMA:GO composites. Raman spectroscopy (Horiba Jobin Yvon, Villeneuve-d’Ascq, France) were separately employed to test the Raman spectra of PMMA:GO nanocomposite films with the proportional chemical compositions of 2:1, 5:1, and 10:1, scanning from 400 cm^−1^ to 4000 cm^−1^. Furthermore, the thermal properties of PMMA:GO was analyzed by TA Instruments (New Castle, DE, USA) under a nitrogen atmosphere at heating rates of 10 °C/min. XRD of PMMA and its blends was tested by an X’Pert PRO XRD meter (Panalytical, Almelo, Finland) with Cu Kα radiation (wavelength = 1.540598 Å). The scans were taken in the 2θ range of 5–30°, with a scan step size of 0.0131303°/mm. UV-vis absorption spectra of PMMA:GO blend solutions were obtained with a U3010 UV-vis spectrophotometer (HITACHI, Tokyo, Japan) in the range of 200–500 nm. Fluorescence spectra were measured with F-4500 FL Spectrophotometer (HITACHI, Tokyo, Japan), using 2.5 nm/2.5 nm slit widths, the response time of 2 s, and the scan speed of 1200 nm/min. The excitation wavelength *λ*_exc_ was 320 nm, and the emission wavelength was read at 350–500 nm. Fluorescence is a novel method to investigate the interaction mechanisms of memristic characteristics based on a GO charge-trap. Memristic characteristics of GO-based resistive switching memory devices were tested by a semiconductor parameter analyzer (Keithley 4200SCS; Keithley, Solon, OH, USA). All measurements of the memory devices without any encapsulation were carried out under ambient conditions. 

## 3. Results

### 3.1. Characterization of GO and PMMA:GO Composite

**Figure 1:**
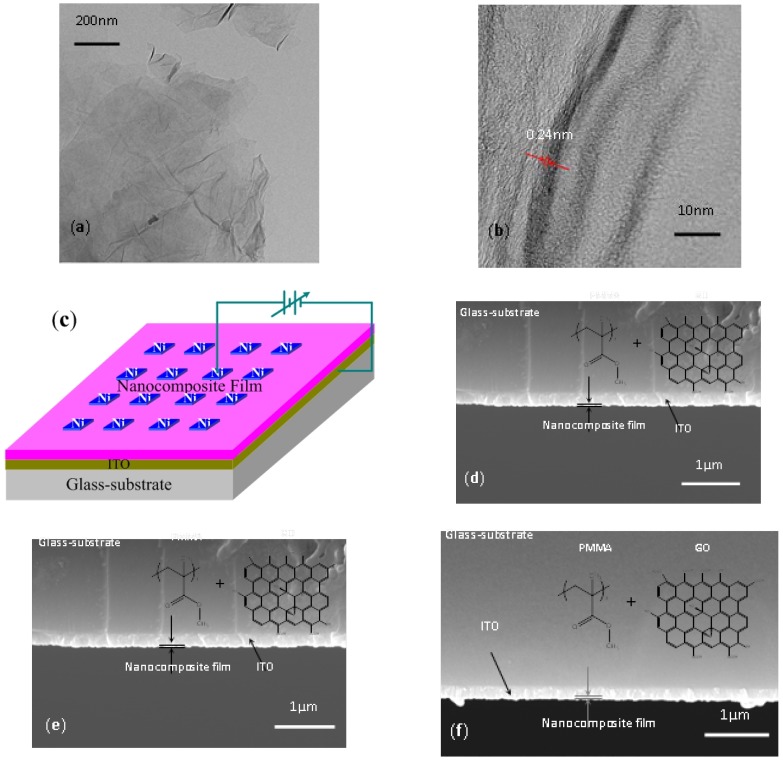
Characterization of GO and the PMMA:GO nanocomposite layer. (**a**) TEM and (**b**) High-resolution TEM micrographs of GO sheets; (**c**) Sandwiched configuration of Ni/PMMA:GO/ITO; (**d**–**f**) SEM images of cross-sectional characterization for PMMA:GO nanocomposite films with chemical component weight ratios of 2:1, 5:1, and 10:1. Among these images, PMMA structure and a schematic representation of GO are shown in the inset.

Figure 1a,b show the characterization of the microstructure in GO, which shows that the GO surface exhibits a wrinkled and aggregated morphology. For observation from the high-resolution transmission electron microscope (HRTEM) image in Figure 1b, the interlayer spacing between GO sheets reaches roughly 0.24 nm. The Ni/PMMA:GO/ITO sandwiched structure is shown in Figure 1c. Figure 1d–f show the cross-sections of PMMA:GO hybrid films, when different amounts of GO are blended with PMMA. The thickness of the nanoscale hybrid films approached 20 nm, as measured by Profilometer. The structure of PMMA and a schematic representation of GO are indicated in the inset of Figure 1d–f. The molecular chain of PMMA is composed of a linear macromolecule methyl methacrylate that can be regarded as a constitutional unit. The chemistry of graphene mainly concentrates on that of GO with chemically reactive oxygen functionality; for example, carboxylic acid groups are present at the edges of GO, and epoxy and hydroxyl groups are present on the basal planes.

**Figure 2:**
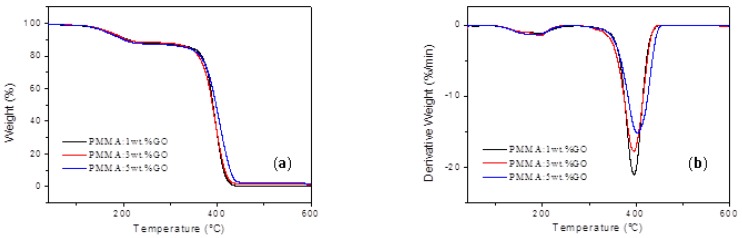
Thermal properties of PMMA: 1 wt % GO, PMMA: 3 wt % GO, and PMMA: 5 wt % GO composite. (**a**) TGA and (**b**) DTG properties of the composites with the content of GO 1 wt %, 3 wt %, and 5 wt %.

**Figure 3:**
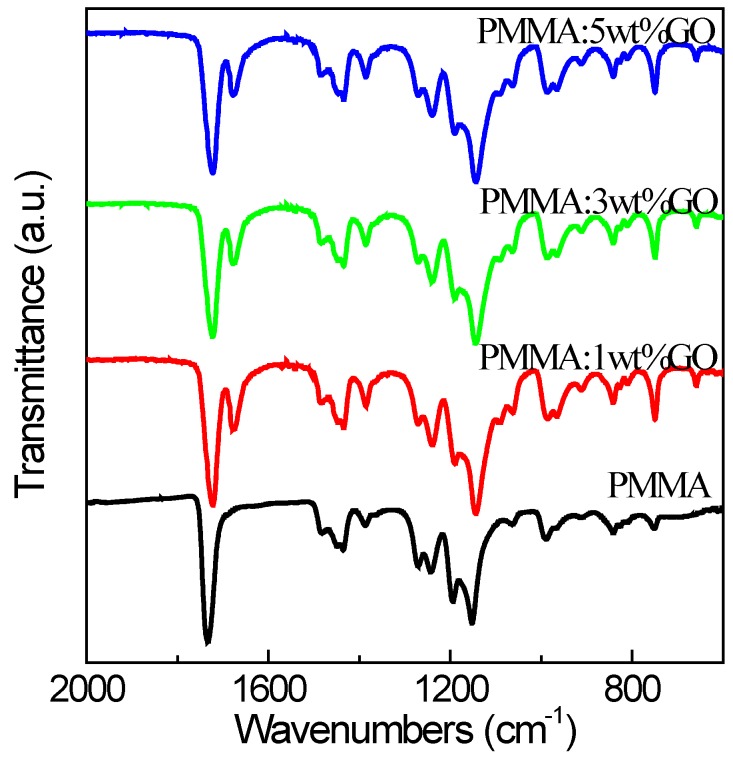
FTIR spectra of PMMA and PMMA:GO composites with the content of GO 1 wt %, 3 wt %, and 5 wt %.

IR spectroscopy of blends was conducted to explore the probable interactions between the binary components. In this study, we performed FTIR spectra for the sake of ascertaining probable interactions between PMMA and GO, as displayed in Figure 2. Figure 3 presents the FTIR spectra of PMMA:GO blends, among which the test wavelength ranged from 600 cm^−1^ to 2000 cm^−1^. The peak at 1144 cm^−1^ owed to the C–O stretching vibration. The O–CH_2_ deformation vibration brings about the obvious peak at 1240 cm^−1^. It should be noted that the C=O stretching vibration contributed to the maximum at 1723 cm^−1^. Figure 3 provides the FTIR spectra of PMMA as well, which demonstrates a variety of bands. The stretching vibrations of C=O and –O–CH_3_ groups of PMMA led to the peaks at 1733 cm^−1^ and 1436 cm^−1^, respectively. The bands at 1484 cm^−1^, 1195 cm^−1^, and 990 cm^−1^ resulted from CH_2_ scissoring, twisting, and wagging modes of PMMA, while the band at 751 cm^−1^ resulted from the CH_2_ rocking mode of PMMA. The band at 1243 cm^−1^ was derived from the C–O stretching vibration of PMMA. The bands at 1387 cm^−1^ and 1152 cm^−1^ stemmed from O–CH_3_ stretching, while the bands at 1678 cm^−1^ and 1271 cm^−1^ came from C=O stretching and CH_2_ stretching vibrations. The distinctive absorption band could be transparently observed at 1195 cm^−1^ because of the C–O stretching of the ester group of PMMA. The stretching absorption band at 1387 cm^−1^ stems from the methyl group of PMMA. For the FTIR spectra of PMMA and PMMA:GO nanocomposites with different chemical composition ratios, the stretching frequency at 1733 cm^−1^ corresponded to the C=O group of PMMA, which was shifted to the higher wavelength side in all of the blends. This shift in the carbonyl-stretching frequencies of the blends may stem from a specific interaction between the carbonyl group of PMMA and the CH_2_ group of GO. It indicates the proper formation of the blends. The incorporation of GO with hydroxyl groups facilitates hydrogen bonding (Figure 1a) with PMMA, which improved the characteristics of the memory devices.

**Figure 4:**
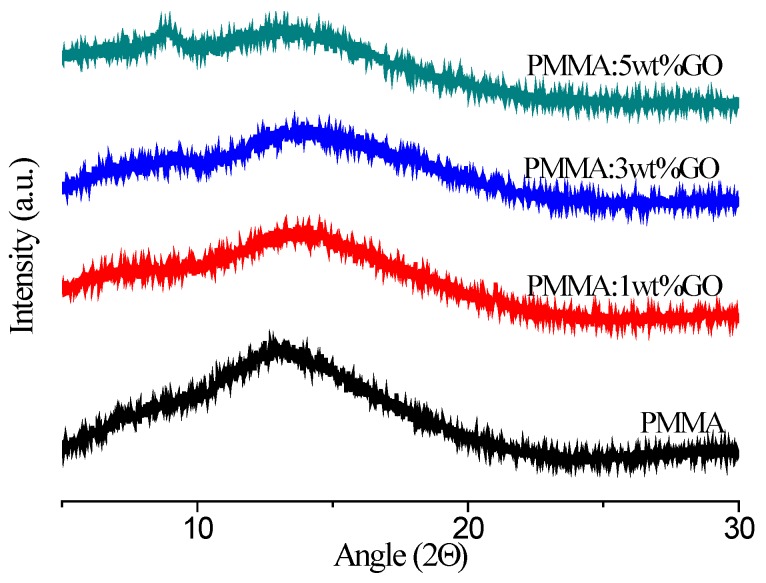
XRD patterns of PMMA and its composites with 1 wt %, 3 wt %, and 5 wt % GO.

XRD is a useful tool to investigate the crystalline forms of PMMA and its blends, as shown in Figure 4. The interplanar distances (*d* values), the angle (2*θ*) values are calculated and listed in Table 1. PMMA bears an amorphous nature that is tested by an amorphous halo (a large hump) at 2*θ* = 13.59° with no sharp peak. Resulting from the absence of a crystalline peak, it is a glassy material. The XRD spectra for PMMA:GO blends in diverse chemical compositions are presented as well. All blend samples exhibited a large hump, which provides a clear indication of the complexity of the blends, due to the fact that the increase in the GO content of the blends contributed to the decrease of the area under the peak. A decrease in the crystallinity was indicated. The d-spacing values decreased from 6.762 Å to 6.262 Å as GO content in the PMMA:GO blend rose, whereas the 2*θ* values grew. Furthermore, the blends possessed a weak peak. Elaborately, the incremental GO content of the blends increased the intensity under the peak, when the d-spacing values decreased from 12.769 Å to 12.018 Å as the GO content increased in the blend, whereas the 2*θ* values increased from 6.917° to 7.350°. The decrease in the *d* value shows that the induction of stress could be understood on the basis of the intermolecular interaction of PMMA blended with GO. Thus, XRD analyses revealed that blending occurred based on the influence of GO on PMMA in the blends.

**Figure 5:**
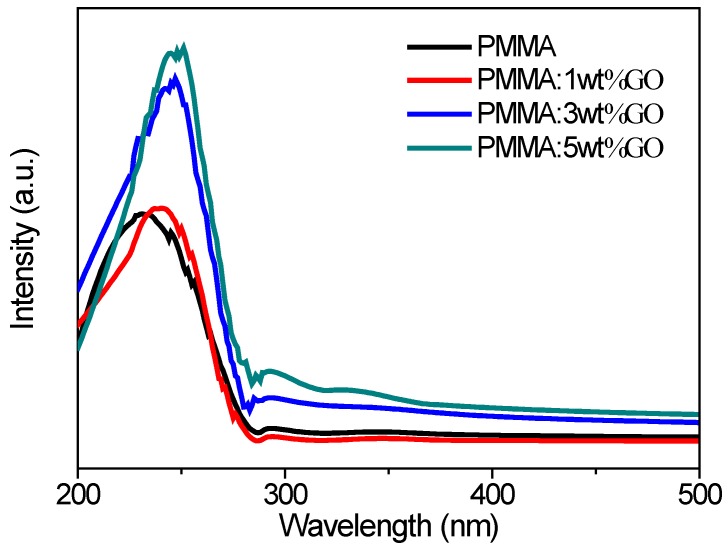
UV-vis absorption spectra of pure PMMA, and PMMA: 1 wt % GO, PMMA: 3 wt % GO, and PMMA: 5 wt % GO composite solutions.

The optical properties of GO-doped polymer solutions were also evaluated by measuring the absorption and fluorescence spectra. UV-vis absorption spectra of PMMA:GO blend dilute solutions (1.25 mg/mL) are shown in Figure 5. Absorption bands were observed in the region of 200–500 nm. The sharp absorption edge for PMMA indicated the semicrystalline nature of PMMA. The maximum absorption peak of the pure PMMA, PMMA: 1 wt % GO, PMMA: 3 wt % GO, and PMMA: 5 wt % GO in DMF was at 231 nm, 240 nm, 247 nm, and 251 nm, respectively. That of the compounds showed a red shift of 9 nm, 7 nm, and 4 nm, respectively. A shift in the absorption band toward a higher wavelength with different absorption intensities occurred for the PMMA:GO blend solutions. These shifts contributed to the intramolecular interactions between PMMA and GO, which were supported by the FTIR spectra and XRD results. Additionally, the absorption edge in the solutions was kept almost the same, at 293 nm, for the optical band gap energy. Clearly, some of the blends exhibited a well-defined window ranging from 200 nm to 350 nm. A sharp and maximum height of this window arose for the blend, with 5 wt % GO. The values of the optical band gap energy *E*_g_ for blends were roughly the same, and they were independent of the GO content.

### 3.2. Memristic Characteristics Based on GO Charge-Trap Memory

**Figure 6:**
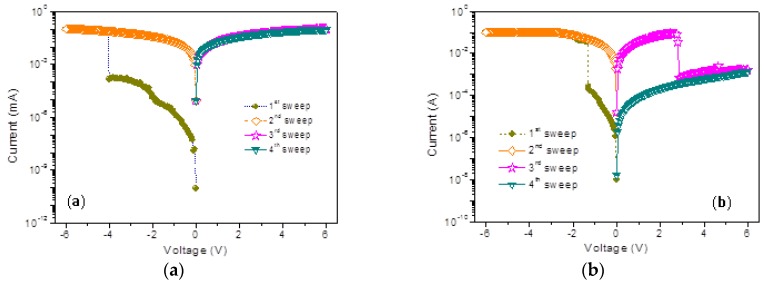

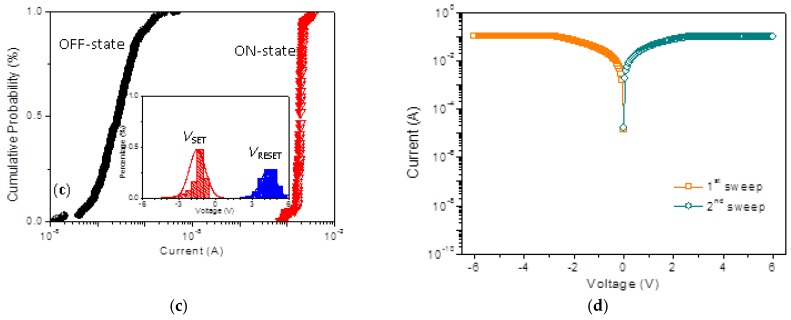
*I*-*V* Characteristics of Ni/PMMA:GO/ITO in terms of different GO weight ratios (**a**) 1 wt %, (**b**) 3 wt %, and (**d**) 5 wt %; (**c**) Cumulative probability of *I*_OFF_ and *I*_ON_ for 300 operation cycles at reading voltage of −0.1 V together with the data distribution of *V*_SET_ and *V*_RESET_.

The memristic characteristics of the PMMA:GO composites are exhibited by the current–voltage (*I*-*V*) curves of the sandwiched Ni/PMMA:GO/ITO memory devices in Figure 6. For the electrical measurements of the memory devices, the Ni top electrodes were grounded, as an electrical bias was applied to the ITO bottom electrodes. Additionally, the compliance current was limited to 10^−1^ A. The *I*-*V* characteristics of the devices manufactured with PMMA:GO as an active layer were initially at low conductivity (OFF-state). Moreover, there was no conductive switching behavior resulting from the insulating nature of PMMA. Blending PMMA with 0.5 wt % GO hardly impacted on device performance switching from the OFF-state, but it produced an increase in electrical conductivity. Furthermore, devices with a GO content of 1 wt % switched from the OFF-state to the ON-state (the high conductivity) when the applied voltage increased from 0 V to −6 V (sweep 1) in Figure 6a. In the subsequent sweep (sweep 2), the device was held in the ON-state, with an ON/OFF state current ratio of above 10^4^ when read at −1 V. At the threshold voltage of 4.1 V, this electrical transition for the memory device was denoted as the “writing” process. After a reverse sweep to +6 V (sweep 3 and sweep 4), the ON-state was kept once it had been turned on, which indicated that the device with 1 wt % GO exhibited write-once-read-many times (WORM) memristic behavior. It displayed both binary and nonvolatile behaviors. The device with 2 wt % GO turned on at a higher voltage of −4 V, and exhibited WORM memory behavior with a lower *I*_ON_/*I*_OFF_. 

The device with 3 wt % GO exhibited electrically rewritable memristic behavior, illustrated by Figure 6b. In initial voltage scanning from 0 V to −6 V, the current, switching from OFF-state to the ON-state, abruptly increased at a threshold voltage of about −1.3 V. The device remained in the ON-state during the subsequent scan from 0 V to −6 V, where the ON/OFF current ratio was more than 10^3^ at a read voltage of −1 V. In the subsequent positive voltage sweep (sweep 3), the device changed from the ON-state to the OFF-state at 2.85 V when scanning from 0 V to 6 V, which was the “erasing process”. Thus it completed a rewritable cycle for a nonvolatile binary memory device. Apart from some minor shifts of the write/erase voltages, 300 operation cycles were repeated with fairly excellent accuracy, shown in Figure 6c. The cumulative probability and data distribution of *V*_SET_ and *V*_RESET_ with normal fitting by Origin 8.0 for 300 cycles were calculated. The minor shift or fluctuation in switching biases may stem from the effect of the electrical stress on the inherent electrical relaxation of the memory materials, and the effect of environmental air and moisture on the electrical properties of the polymer/metal interface. The growth of GO content results in an incremental conductivity of the composite films, and devices with more than 5 wt % GO all exhibited conductor behavior, as shown in Figure 6d. This GO-blended strategy principally prompts diverse fascinating intermediates from the insulator to the conductor.

**Figure 7:**
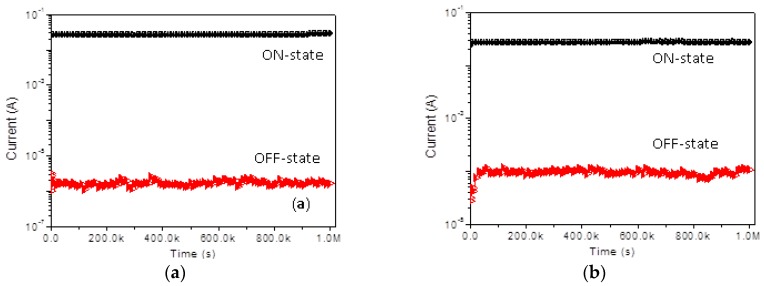
Retention ability of *I*_ON_ and *I*_OFF_ for (**a**) Ni/PMMA: 1 wt % GO/ITO and (**b**) Ni/PMMA: 3 wt % GO/ITO.

As for the retention ability at *V* = −1 V, *I*_ON_/*I*_OFF_ of above 10^4^ was achieved in WORM memory devices, while that above 10^3^ was gained in rewritable memory devices. No significant degradation of the device in both the ON-state and the OFF-state occurred during the continuous stress time of 10^6^ s, which indicates that the composite materials and the electrode/polymer interfaces are both stable. The ON/OFF current ratio in the bistable devices was sufficiently high to promise a low misleading rate. The effect of continuous read pulses with *V*_read_ = −1 V on in the ON-state and OFF-state was also investigated. As shown in Figure 7a,b, more than one million read cycles were performed on the Ni/PMMA:GO/ITO devices, and no current degradation was observed for the ON-state and the OFF-state. Neither the voltage stress nor the read pulse caused conductive state variation where the applied voltage (−1 V) is lower than the threshold voltage. Both the ON-state and OFF-state, therefore, were stable under voltage stress and are immune to read pulses.

**Figure 8:**
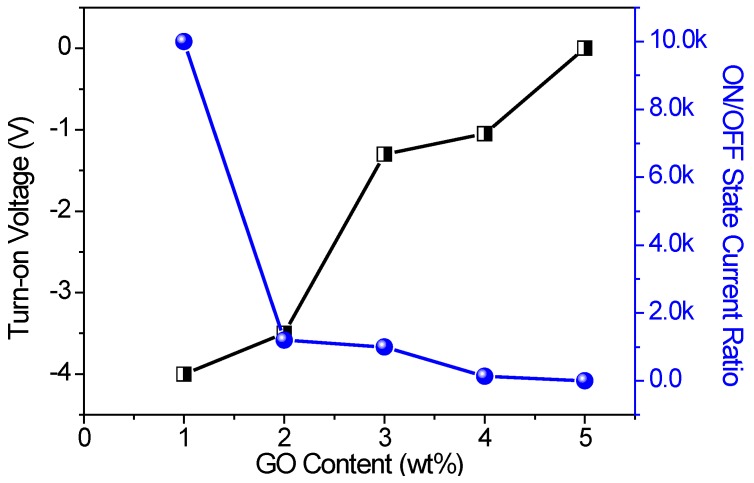
Effect of the GO content on the turn-on voltage and the ON/OFF state current ratio in memristic performance.

As for TEM, HRTEM, and SEM images of Figure 1, GO was uniformly distributed in the PMMA matrix. GO could be well integrated into the polymer matrix. The effect of the GO content on memristic performance included the turn-on voltage and the ON/OFF state current ratio, as summarized in Figure 8. *I*_ON_/*I*_OFF_ of the nanocomposite films was enhanced by four orders magnitude with the incremental GO content from 1 wt % to 5 wt %. The turn-on voltage for the memory devices grew from −4 V to −1.05 V, while the GO content increased from 1 wt % to 4 wt %.

### 3.3. Operation Mechanism of Resistive Switching based on Memristic Characteristics

**Figure 9:**
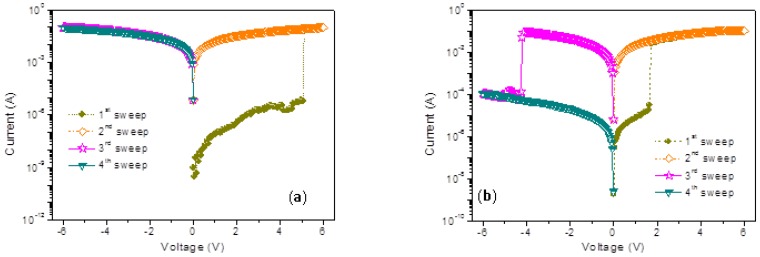
Bipolar tunable memristic behaviors for (**a**) Ni/PMMA: 1 wt % GO/ITO and (**b**) Ni/PMMA: 3 wt % GO/ITO.

The work function of Ni (−5.1 eV) is higher than that of ITO (−4.8 eV), leading to a higher energy barrier for electron injection from the electrode into the PMMA matrix. Electrical measurements were also implemented by sweeping from the positive voltage at first, so that *I*-*V* characteristics of ITO/PMMA:GO/Ni device exhibited bistably memristic behaviors, similar to those of ITO/PMMA:GO/Ni device, by sweeping from the first negative voltage. Therefore, the device with 1 wt % GO performed WORM memory, while the device with 3 wt % GO exhibited rewritable memory. Exhibited in Figure 9, a higher absolute turn-on voltage is required in the ITO/PMMA:GO/Ni devices, and were 5.05 V and 1.65 V for Ni/PMMA: 1 wt % GO/ITO and Ni/PMMA: 3 wt %GO/ITO, respectively. Furthermore, the *I*-*V* characteristics of the PMMA:GO nanocomposite films were independent of the metallic filamentary, ruling out the possibility of random metallic filamentary conduction, which might give rise to electrical bistability. Obviously, the *I*-*V* characteristics of the PMMA:GO nanocomposite films strongly relied on the GO content. The memristic behaviors, therefore, must be intrinsic to PMMA:GO composites. 

**Figure 10:**
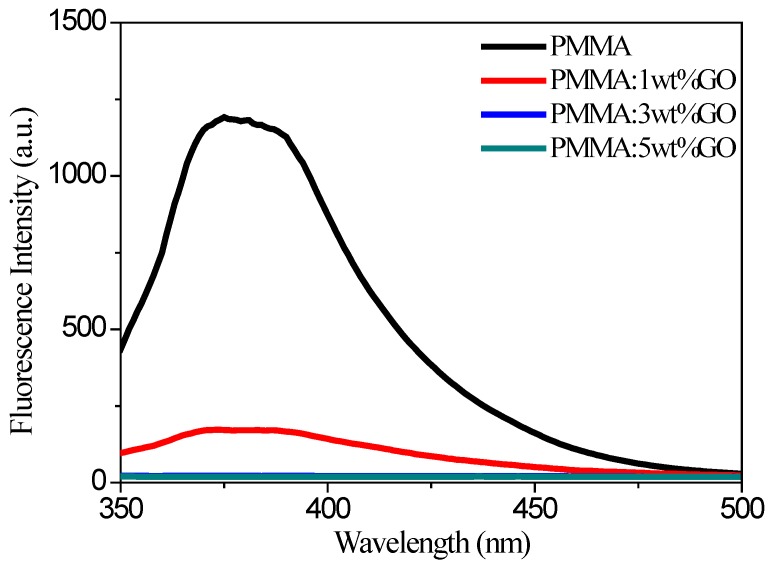
Fluorescence emission spectra excited at 320 nm for pure PMMA solutions, as well as the composite solution with a GO content of 1 wt %, 3 wt %, or 5 wt %.

Fluorescence spectra for pure PMMA and PMMA:GO blends were carried out as shown in Figure 10. The incremental quencher GO content led to fluorescence quenching. The pure PMMA in DMF solution showed maximum absorption at 375 nm. For PMMA:GO blend solutions, there was a blue shift of 1 nm for the fluorescence emission spectra, compared with the DMF solution of PMMA. Photochromatic PMMA exhibited high sensitivity to oppositely charged molecular quenchers, which was anticipated to quench fluorescence by electron or energy transfer. Quenching was depicted for DMF solutions of PMMA:GO, and attributed to a combination of a strong relationship between PMMA and GO with energy migration and/or delocalization within PMMA until quenching arose at trap sites where quencher GO connects with one or more polymer repeat units [22]. The strong association between organic and inorganic molecules is derived from a combination of Coulombic and hydrophobic interactions. From an investigation into fluorescence spectra, the memristic performance mechanism of PMMA:GO blends influence the GO charge-trap memory.

**Figure 11:**
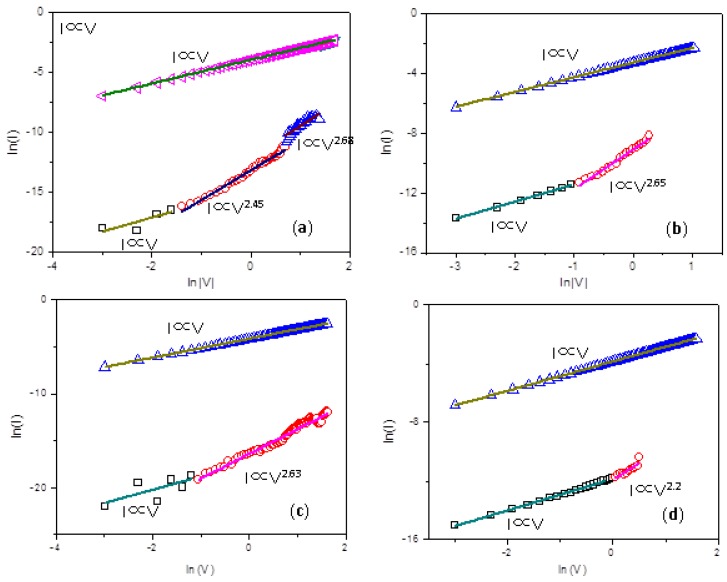
Linear fitting for *I-V* curve of on a log-log scale firstly during a negative voltage sweep for (**a**) Ni/PMMA: 1 wt % GO/ITO and (**b**) Ni/PMMA: 3 wt % GO/ITO; Linear fitting for the *I*-*V* curve of on a log-log scale during a positive voltage sweep for (**c**) Ni/PMMA: 1 wt % GO/ITO and (**d**) Ni/PMMA: 3 wt % GO/ITO.

To understand the charge transport of the PMMA:GO films, the nonlinear *I*-*V* curves in the origin sweep from the negative scan and positive scan were plotted in a log-log scale, as indicated in Figure 11. The *I*-*V* relationship in the ON-state clearly had Ohmic conduction, with a slope of 1, which was ascribed to the formation of conducting paths during the writing process. Nevertheless, the conducting behavior in the OFF-state was much more complicated. The fitting results for OFF-state showed charge transport behavior similar to space charge-limited conduction (SCLC). It consists of three different regions, an Ohmic region (*I* ∝ *V*), a Child’s law region (*I* ∝ *V*^α^, *α* > 2), and a sharp current increase region. The totally different conduction behaviors were due to a localized conducting effect rather than a homogeneously distributed one.

Concerning the charge transport mechanism, further information can be obtained from the *I*-*V* curves in the OFF-state and the ON-state. Taking the low content of GO in the composites into account, PMMA is the dominant component, and it can be regarded as the matrix of the active layer. GO is more likely to serve as the electron trapping center and electron transporter [19,20,21]. When the negative bias (Ni as cathode) is applied, electrons are injected into the nanocomposites and trapped by GO. The trapped electrons can induce a countering space–charge layer in the PMMA neighboring the Ni electrode. At a low bias, electrons do not bear sufficient energy or mobility to escape from the isolated GO trapping centers surrounded by the PMMA matrix. In the OFF-state of bistable devices, *I*-*V* curves can be fitted by SCLC. For memory devices with 0.5 wt % graphene, the large separation between the GO sheets prevents the charge carriers from interplane hopping, although charge carriers can acquire activation energy from the external electric field, and their mobility rises with the incrementally applied voltage. Thus, the above device always performs within a single low-conductivity state, and no memristic characteristics can be observed. With increasing GO content in the composites, the distance between the isolated GO sheets are reduced. At the threshold switching voltage, most of the charge-trapping centers are filled, so that a trap-free environment exists in the composite film. Percolation pathways for charge carried among PMMA:GO nanocomposite films are formed, allowing for interplane hopping and switching of devices from the OFF-state to the ON-state. *I*-*V* curves for the ON state currents for WORM memory devices can be fitted by a combination of the space-limited model and the Ohmic model. With a further incremental content in GO, GO in the composite thin film may eliminate the space charge layer formed near the PMMA/Ni interface. *I*-*V* curves of the devices with 3 wt % GO are predominantly consistent with SCLC model, for which the traps are exponentially distributed within *E*_g_ of PMMA, and the formation of the space charges is obviously relevant to the carriers trapped in GO. This brings about the writing process. When the applied positive bias is more than *V*_RESET_, the electrons are detrapped in GO, and the conducting paths in the PMMA:GO film are destroyed close to the electrode, leading to the erasing process [21]. For memory devices with 5 wt % GO, continuous networks are formed in the bulk film, which is responsible for the effective transport of charge carriers, even under the low bias. That makes the devices highly conductive. 

## 4. Conclusions

The ITO/PMMA:GO/Ni structure that is capable of exhibiting bistably bipolar memristic characteristics, is demonstrated. Electrical conductance behaviors, turn-on voltage, and ON/OFF state current ratio, can be tuned through the control of the GO content in the composites. Under ambient conditions, both the OFF-state and ON-state of the bistable memory devices are stable under a constant voltage stress. The conductance switching effects of the composites can be attributed to electron trapping in GO sheets. With the benefits of its solution processability and good performance, the PMMA:GO composite memory device is potentially useful for high capacity and low-cost data storage in electronics in the future.

## Figures and Tables

**Table 1:** XRD results for PMMA and its nanocomposites.

PMMA:GO Blend	2*θ*	*d* (Å) ^a^	2*θ*	*d* (Å) ^a^
PMMA	13.082	6.762	-	-
PMMA: 1 wt % GO	14.056	6.295	6.917	12.769
PMMA: 3 wt % GO	14.424	6.136	7.720	11.442
PMMA: 5 wt % GO	14.132	6.262	7.350	12.018

^a^*d* = *nλ*sin*θ*, where *λ* is the wavelength, *n* is an integer determined by the order given, and *θ* is the angle between the incident ray and the scattering planes.
